# Effect of vitamin D supplementation on cardiovascular outcomes: an updated meta-analysis of RCTs

**DOI:** 10.1097/MS9.0000000000002458

**Published:** 2024-08-14

**Authors:** Agha M.W. Mirza, Naiela E. Almansouri, Muhammad F. Muslim, Thahaseen Basheer, Sree V. Uppalapati, Swati Lakra, Hareem Fatima, Arshiya Adhnon, Ivo W. Filho, Ruqeyya Mahmood, Mahendra Kumar, Kamal Kandel, Muhammad Ayyan

**Affiliations:** a Department of Internal Medicine, Jinnah Medical & Dental College, Karachi; b Department of Internal Medicine, Mayo Hospital; c Department of Internal Medicine, Allama Iqbal Medical College, Lahore; d Department of Internal Medicine, Federal Medical College, Islamabad, Pakistan; e Department of Internal Medicine, Karuna Medical College, Kerala; f Department of Internal Medicine, Holy Heart Hospital, Rohtak, Haryana; g Department of Internal Medicine, Sardar Patel Medical College, Bikaner, Rajasthan; h Department of Internal Medicine, Deccan College of Medical Sciences, Hyderabad, Telangana, India; i Department of Internal Medicine, Faculdade Pernambucana de Saúde (FPS), Recife, Brazil; j Department of Internal Medicine, Dubai Medical College for Girls, Dubai, United Arab Emirates; k Department of Internal Medicine, All American Institute of Medical Sciences, Jamaica; l Department of Medicine, Kathmandu University, Kathmandu, Nepal; m Department of Internal Medicine, University of Tripoli Faculty of Medicine, Tripoli, Libya

**Keywords:** cardiology, cardioprotective, cardiovascular events, cardiovascular, meta-analysis, vitamin D

## Abstract

**Objective:**

To evaluate the effect of vitamin D supplementation on cardiovascular outcomes.

**Methods:**

After searching different databases, we retrieved and included randomized controlled trials on long-term supplementation of vitamin D (≥1-year intervention) and reporting cardiovascular outcomes. We calculated risk ratio (RR) with 95% confidence intervals (CI) for dichotomous outcomes.

**Results:**

Compared to the control group, the vitamin D group was not associated with a statistically significant decrease in the incidence of major adverse cardiovascular events (MACE) [risk ratio=0.99; 95% CI: 0.94–1.03]. We found no difference between the vitamin D group and the control group for the outcomes of incidences of myocardial infarction, heart failure, coronary revascularization, cardiovascular death, and all-cause mortality. The heterogeneity was low for all outcomes.

**Conclusion:**

According to our meta-analysis, vitamin D supplementation did not reduce major adverse cardiovascular events, other cardiovascular parameters, and all-cause mortality.

## Introduction

Highlights

- Compared to the control group, the vitamin D group was not associated with a statistically significant decrease in the incidence of major adverse cardiovascular events (MACE).
- We found no difference between the vitamin D group and the control group for the outcomes of incidences of myocardial infarction, heart failure, coronary revascularization, cardiovascular death, and all-cause mortality.

Vitamin D deficiency (VDD) is prevalent worldwide, with 15.7% of individuals having serum 25-hydroxyvitamin D levels below 30 nmol/l^1^. Cardiovascular disease (CVD) is a significant public health issue and the primary contributor to mortality globally, with around 17.7 million deaths attributed to CVD^2^. CVD development is influenced by a multitude of factors. Among the most significant contributors to CVD is the nutritional component^3^.

VDD and cardiovascular ailments have become quite prevalent, with both conditions commonly occurring together^4^. Numerous observational studies^5,6^ have shown a correlation between vitamin D deficiency and an elevated risk of cardiovascular disease and overall mortality. In the same manner, multiple studies have examined the correlation between vitamin D deficiency and coronary artery diseases (CADs)^7–9^. Approximately 95% of patients admitted with acute coronary syndrome (ACS) in a cohort study exhibited low vitamin D levels^10^. Similarly, Dziedzic *et al.* ^11^ conducted a study that revealed that patients with a history of myocardial infarction had low levels of vitamin D.

However, many randomized clinical trials have not been able to establish any conclusive connection between vitamin D supplementation and a decrease in cardiovascular outcomes like major adverse cardiovascular events^12,13^. It should be noted that these trials were carried out on diverse subject groups without taking into account their baseline levels of 25(OH)D concentration^14^.

A total of five meta-analyses have been published to assess the impact of vitamin D supplementation on cardiovascular mortality^15–19^. Three meta-analyses^15–17^ included trials that administered both vitamin D and calcium together, making it difficult to determine the individual effect of vitamin D. All of the meta-analyses have reported that there is no cardiovascular benefit of vitamin D supplementation. Hence, the US Preventive Services Task Force has not recommended the administration of vitamin D to prevent cardiovascular disease. Our meta-analysis is an updated analysis because it includes a recently published large-scale RCT with 21 315 participants^20^. Thus, we carried out an updated meta-analysis of RCTs that evaluate the efficacy of vitamin D supplementation for preventing cardiovascular disease.

## Materials and methods

Our meta-analysis was conducted according to the guidelines presented in the *Cochrane Handbook for Systematic Reviews of Interventions* and followed the reporting guidelines described in the Preferred Reporting Items for Systematic Reviews and Meta-Analysis (PRISMA, Supplemental Digital Content 1, http://links.lww.com/MS9/A581) statement^21,22^. Our study did not require any ethical approval. This meta-analysis has been registered with the International Prospective Register of Systematic Reviews (PROSPERO). This review has been reported in line with the AMSTAR criteria (Supplemental Digital Content 2, http://links.lww.com/MS9/A582).

### Eligibility criteria

The inclusion criteria were as follows: (1) only RCTs; (2) adults who received vitamin D in various forms and doses, with or without concurrent calcium administration; (3) studies with at least one cardiovascular outcome reported; (4) studies on only long-term supplementation (≥1-year intervention) of vitamin D. Studies that did not include cardiovascular outcomes or with an intervention period <1 year were excluded. We also excluded observational studies and review studies.

### Information sources

We searched electronic databases, gray literature sources, and international trial registries from their inception until September 1, 2023, without imposing any language restrictions. The databases and registries searched included the Cochrane Central Register of Controlled Trials (accessed via the Cochrane Library), MEDLINE (via PubMed), Embase (via Ovid), ClinicalTrials.gov, and the World Health Organization International Clinical Trials Registry Platform portal. The reference lists of the included studies and relevant meta-analyses were also screened to find other RCTs. A search strategy consisting of a combination of keywords and MeSH terms like vitamin D, ergocalciferol, cholecalciferol, cardiac, myocardial, and cardiovascular were used.

### Study selection and data extraction

The retrieved articles from the search were imported into Mendeley Desktop 1.19.8, and any duplicate articles were removed. Two review authors then conducted a two-step process to screen studies: first, by assessing titles and abstracts, and secondly, by reviewing the full-text versions of selected studies. Any disagreements during this process were resolved through discussion, with a plan in place for a third review author to serve as an arbitrator if needed. A PRISMA flow chart was used to visually present the selection process.

Following the completion of the study selection, two reviewers extracted relevant data from selected studies using a standardized data extraction form. This information encompassed various aspects, including study details (such as design, location, year of publication, and author), participant characteristics (including number of participants, age range, and gender distribution), intervention specifics (dosage, duration, and follow-up period), baseline parameters, comparator, and outcome measures assessed.

### Outcome measures

The primary outcome is the incidence of major adverse cardiovascular events (MACE). The secondary outcomes included: (1) all-cause mortality, (2) incidence of cardiovascular death, (3) incidence of MI, (4) incidence of cerebrovascular events, (5) incidence of HF, and (6) incidence of coronary revascularization.

### Risk of bias assessment

We assessed the risk of bias in the included studies using the revised Cochrane “Risk of bias” tool for randomized trials (RoB 2.0), which assesses bias in five domains: (1) bias arising from the randomization process, (2) bias due to deviations from intended interventions, (3) bias due to missing outcome data, (4) bias in the measurement of the outcome, and (5) bias in the selection of the reported result^23^. Two authors independently assessed the risk of bias in each study that was included, categorizing it as either low-risk, high-risk or have some concerns. In cases where there were discrepancies between the assessments of the two original authors, a third reviewer was consulted to help make a final decision.

### Data synthesis

For each trial, we reported dichotomous outcomes as relative risk (RR) along with 95% confidence intervals. The meta-analysis was conducted when at least two studies reported the relevant outcome data. We used the DerSimonian and Laird random-effects model in our meta-analyses. For detection and quantification of heterogeneity, we calculated the *χ* ^2^ test and *I* ^2^ statistic. We interpreted *I* ^2^ values according to the Cochrane Handbook for Systematic Reviews of Interventions section 10.10. *P* value <0.10 was considered statistically significant for the *χ* ^2^ test^24^. All statistical analyses were performed using Review Manager (RevMan, Version 5.4; The Cochrane Collection, Copenhagen, Denmark). The study characteristics and findings of the included studies were presented in the form of tables.

## Results

**Figure 1:**
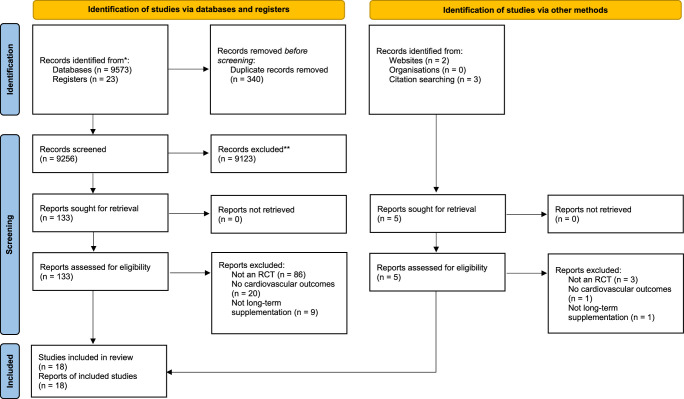
PRISMA 2020 flow chart. Flow chart of included and excluded trials. PRISMA, Preferred Reporting Items for Systematic Reviews and Meta-Analyses.

After a comprehensive database search yielding 9596 records with five records identified through other sources, we screened the articles, and 18 RCTs were included in our meta-analysis^12,13,20,25–39^. The PRISMA flow chart is presented in Figure 1. Seven studies included only postmenopausal women, while other studies included older patients, patients on hemodialysis, and patients with heart failure. The follow-up period of the studies ranged from 1 to 6 years. Out of the 18 RCTs, 15 trials used cholecalciferol (vitamin D3), two trials used ergocalciferol^31,33^, and one trial^38^ used alfacalcidol as the intervention. Cumulatively 108 385 participants are included in this meta-analysis, with 54 636 in the vitamin D group and 53 749 in the control group. The mean age was found to be 67.68 years. The study characteristics are presented in Table 1.

**Table 1:** Characteristics of included studies

Study ID	Country	Number of patients	Study period	Follow-up period (years)	Vitamin D type and dosage	Age mean (SD) (vitamin D vs. placebo)	Male n (vitamin D vs. placebo)	Current smoker no. (vitamin D vs. placebo)	BMI, mean (SD) (vitamin D vs. placebo)	Total cholesterol, mean (SD), mg/dl (vitamin D vs. placebo)	Baseline 25-hydroxyvitamin D level, mean (SD), ng/ml (vitamin D vs. placebo)	Statin user, No. (vitamin D vs. placebo)	Systolic BP, mmHg, mean (SD) (vitamin D vs. placebo)	HTN, no. (vitamin D vs. placebo)	DM, no. (vitamin D vs. placebo)
Aloia *et al*.^25^	United States	27	NA	2 y	Vitamin D3 400 IU/d	64.1 (1.5) vs. 64.9 (1.7)	0 vs. 0	NA	NA	NA	21.9 (7) vs. 26.6 (12)	NA	NA	NA	NA
Baron *et al*.^28^	United States	2259	4 y	3 y	Vitamin D3 1000 IU /d	58.3 (7.0) vs. 58.2 (7.0)	358 vs. 355	39 vs. 35	29.1 (4.6) vs. 29.0 (4.9)	NA	24.88 (8.09) vs. 24.24 (7.84)	NA	NA	NA	NA
Chatterjee *et al*.^36^	United States	2385	5 y	2.9 y	Vitamin D3 400 IU/d	59.6 (9.8) vs. 60.4 (10.0)	663 vs. 660	74 vs. 79	32 (4.5) vs. 32.1 (4.4)	NA	27.8 (10.2) vs. 28.2 (10.1)	511 vs. 501	NA	NA	423 vs. 423
D-Health Trial	Australia	21315	6 y	5 y	Vitamin D3 60000 IU/mo	NA	5765 vs. 5760	NA	NA	NA	NA	3769 vs. 3681	NA	4483 vs. 4368	NA
EVITA/Zitterman *et al*.	Germany	400	3 y	2.7 y	Vitamin D3 400 IU/d	55.5 (4.0) vs. 54 (3.5)	166 vs. 166	NA	27.9 (2.0) vs. 27.9 (1.6)	NA	32.2 (6.7) vs. 36.3 (6.8)	113 vs. 105	115.5 (7.5) vs. 117 (6.9)	57 vs. 63	51 vs. 46
J-DAVID/Shoji *et al*.	Japan	964	3 y	4 y	Oral alfacalcidol 0.5 μg/d	64.75 (3.7) vs. 64.6 (3.5)	301 vs. 277	NA	21.15 (1.3) vs. 21.1 (1.2)	153.2 (12.4) vs. 150 (11.5)	NA	77 vs. 81	145 (8.7) vs. 147.5 (7.5)	NA	207 vs. 204
Komulainen *et al*.^27^	Finland	225	8 y	5 y	Vitamin D3, 300 IU/d, lowered to 100 IU/d in 5th year	52.87 (0.3) vs. 52.6 (0.3)	0 vs. 0	NA	27.1 (0.5) vs. 26.5 (0.5)	NA	28.1 (2.8) vs. 28.0 (2.5)	NA	NA	NA	2 vs. 3
Ott *et al*.^26^	United States	86	NA	2 y	Vitamin D3, 0.25 μg capsules (2 per day in the start and then increased by 1 or 2 capsules/day later in the study	67.9 (1.0) vs. 67.1 (1.2)	0 vs. 0	NA	NA	NA	26.7 (1.9) vs. 26.3 (2.4)	NA	NA	NA	NA
RECORD/Grant *et al*.^37^	United Kingdom	5292	3 y	5.2 y	Vitamin D3, 800 IU/d	77 (6) vs. 77 (6)	15 vs. 15	299 vs. 320	NA	NA	NA	NA	NA	NA	NA
Schleithoff *et al*.^30^	Germany	123	1 y	1.3 y	Vitamin D3, 2000 IU/d	57.6 (7.5) vs. 53.3 (9)	52 vs. 50	9 vs. 7	26.3 (3.8) vs. 26 (3.1)	NA	16.1 (2.9) vs. 16.1 (2.9)	53 vs. 42	120 (6) vs. 125 (8)	38 vs. 32	20 vs. 23
STOP IT/Gallagher *et al*.^31^	United States	489	NA	3 y	Calcitriol 0.5 μg/d	72 (3) vs. 71 (4)	0 vs. 0	NA	NA	NA	78.0 (21.6) vs. 80.5 (27.4)	NA	NA	NA	NA
Trivedi *et al*.^32^	United Kingdom	2686	6 y	5 y	Vitamin D3, 100 000 IU/4 mo	74.8 (4.6) vs. 74.7 (4.6)	1019 vs. 1018	59 vs. 53	24.3 (3.4) vs. 24.4 (3.0)	NA	NA	NA	NA	NA	NA
VIDA/Scragg *et al*.^13^	New Zealand	5108	1 y	3.3 y	Vitamin D3 200000 IU initial bolus dose then 100000 IU(2.5 mg) capsule	65.9 (8.3) vs. 65.9 (8.3)	1512 vs. 1457	164 vs. 156	28.4 (5.1) vs. 28.5 (5.1)	185 (42) vs. 189 (42)	24.4 (9.6) vs. 24.4 (9.6)	NA	139 (19) vs. 139 (19)	34 vs. 40	265 vs. 239
Virtanen *et al*.^29^	Finland	2495	6 y	5 y	Vitamin D3 1600 IU/d	68.2 (4.4) vs. 68.2 (4.5)	968 vs. 458	593 vs. 292	27.0 (4.3) vs. 27.2 (4.3)	NA	NA	462 vs. 255	NA	694 vs. 353	154 vs. 68
Vital D/Sanders *et al*.^34^	Australia	2256	2 y	2.96 y	Vitamin D3, 500 000 IU/y	76.4 (5.7) vs. 76.5 (5)	0 vs. 0	NA	NA	NA	53 (7) vs. 47 (5)	NA	NA	NA	NA
VITAL/Manson *et al*.	United States	25871	3 y	5.3 y	Vitamin D3 2000 IU/d	67.1 (7.0) vs. 67.1 (7.1)	6380 vs. 6406	921 vs. 915	28.1 (5.7) vs. 28.1 (5.8)	NA	NA	4822 vs. 4702	NA	6352 vs. 6439	1812 vs. 1737
WHI/Jackson *et al*.^39^	United States	36282	10 y	7 y	Vitamin D3, 400 IU/d	62.4 (7.0) vs. 62.4 (6.9)	0 vs. 0	1405 vs. 1356	29.1 (5.9) vs. 29.0 (5.9)	208.1 vs. 208.1	NA	1178 vs. 1149	127 (17) vs. 128 (17)	5447 vs. 5476	1055 vs. 1036
Zhu *et al*.^33^	Australia	120	1 y	5 y	Vitamin D2, 1000 IU/d	75.4 (2.7) vs. 74.4 (2.4)	0 vs. 0	NA	27.6 (4) vs. 28.2 (4.1)	NA	26.8 (10.4) vs. 28 (10.4)	NA	NA	NA	NA

### Incidence of MACE

**Figure 2:**
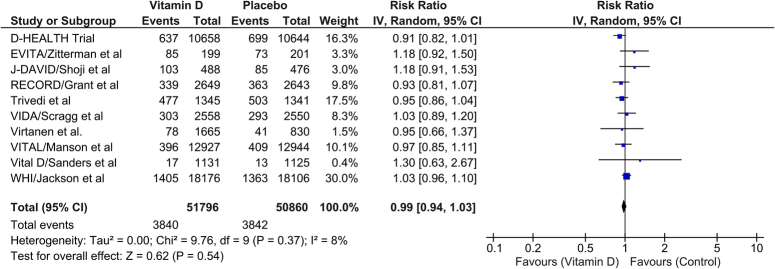
Comparison of incidence of MACE between patients receiving vitamin D or control. IV, inverse variance. MACE, major adverse cardiovascular events.

A total of 10 RCTs reported the incidence of major adverse cardiovascular events (MACE). According to our pooled data, the vitamin D intervention was not associated with a statistically significant decrease in the incidence of major adverse cardiovascular events, with the risk ratio being 0.99 (95% CI: 0.94–1.03). The heterogeneity between studies was found to be low (*I* ^2^=8%) for this outcome (Fig. 2).

### All-cause mortality

**Figure 3:**
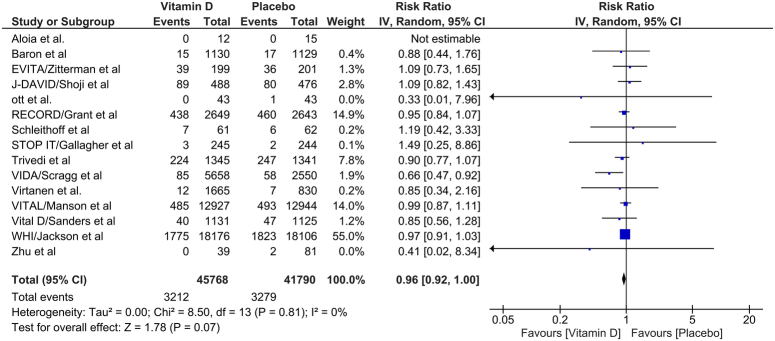
Comparison of all-cause mortality between patients receiving vitamin D or control. IV, inverse variance.

The all-cause mortality was reported by 15 RCTs in our meta-analysis. We found no difference in the all-cause mortality between the two groups in our meta-analysis [RR 0.96; 95% CI: 0.92–1.00]. The heterogeneity between studies was found to be low (*I* ^2^=0%) (Fig. 3).

### Cardiovascular death

**Figure 4:**
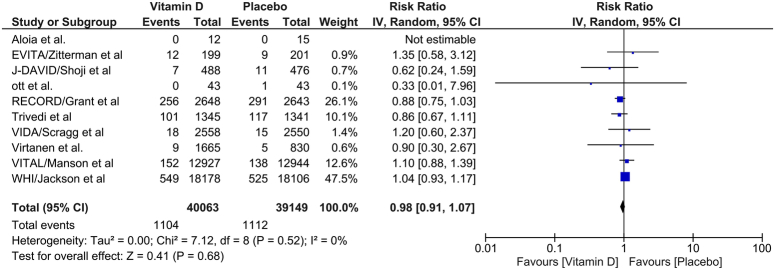
Comparison of incidence of cardiovascular death between patients receiving vitamin D or control. IV, inverse variance.

A total of 10 RCTs reported the outcome of cardiovascular death. Vitamin D intervention was not associated with a statistically significant decrease in cardiovascular death [RR 0.99; 95% CI: 0.91–1.07]. The heterogeneity between studies was found to be low (*I* ^2^=0%) for this outcome (Fig. 4).

### Incidence of MI

The incidence of MI was reported by 14 RCTs in our meta-analysis. We found no difference in the incidence of MI between the two groups in our meta-analysis [RR 0.97; 95% CI: 0.90–1.04]. The heterogeneity between studies was found to be low (*I* ^2^=0%) (Supplementary Fig. S1.1, Supplemental Digital Content 3, http://links.lww.com/MS9/A583).

### Incidence of cerebrovascular accident

According to our meta-analysis of 12 studies reporting this outcome, vitamin D did not reduce the incidence of cerebrovascular accidents [RR 1.04; 95% CI: 0.97–1.12; *I* ^2^=0%] (Supplementary Fig. S1.2, Supplemental Digital Content 3, http://links.lww.com/MS9/A583).

### Incidence of heart failure

Only two studies reported this outcome, and the meta-analysis of these two studies showed no statistically significant difference between vitamin D and control groups [RR 1.17; 95% CI: 0.86–1.60; *I* ^2^=0%] (Supplementary Fig. S1.3, Supplemental Digital Content 3, http://links.lww.com/MS9/A583).

### Incidence of coronary revascularization

Only three studies reported this outcome and the meta-analysis of these three studies showed no statistically significant difference between vitamin D and control groups [RR 0.91; 95% CI: 0.80–1.03; *I* ^2^=0%] (Supplementary Fig. S1.4, Supplemental Digital Content 3, http://links.lww.com/MS9/A583).

### Risk of bias of included studies

After assessing each study for the risk of bias using the Rob 2.0 tool, 14 out of 18 studies were judged to be at low risk of bias. Four studies were judged to be at medium risk of bias due to concerns related to attrition bias, allocation concealment, and prespecified statistical plan.

The complete risk of bias assessment is presented in Supplementary Figure S1.5. (Supplemental Digital Content 3, http://links.lww.com/MS9/A583).

## Discussion

According to our meta-analysis of 18 RCTs on 108 385 patients, vitamin D was not associated with a statistically significant decrease in the incidence of major adverse cardiovascular events (MACE), all-cause mortality, cardiovascular death, incidence of heart failure, myocardial infarction, cerebrovascular accident, and coronary revascularization. It is noteworthy to note that the evidence was not sufficient on the outcomes of cerebrovascular accident and coronary revascularization rates. Some previous observational studies^5,6^ showed a potential effect in reducing adverse cardiovascular events but no such association has been observed in many RCTs and previous meta-analyses^18,19^ conducted on vitamin D effect on cardiovascular outcomes. Our meta-analysis includes data from a very large randomized controlled trial, the D-Health trial^20^, which was not included in the previous meta-analyses.

Our meta-analysis’ findings are similar to the results of the previous meta-analyses. Barbarawi *et al.* ^18^ reported that vitamin D did not significantly reduce major adverse cardiovascular events [RR 1.00; 95% CI: 0.95–1.06]. The effect on myocardial infarction, stroke, CVD mortality, and all-cause mortality was also insignificant. Pei *et al.* ^19^ conducted a meta-analysis on 18 RCTs, and they showed a similar effect on all cardiovascular outcomes, including MACE, myocardial infarction, heart failure, and cerebrovascular events. Our meta-analysis is different from the previous meta-analyses in two ways: (1) in this study, we have only included RCTs on long-term (≥1-year intervention) vitamin D supplementation; (2) inclusion of a large trial, the D-Health Trial. In spite of including more trials, our findings are similar to the previous meta-analyses. This may suggest that it is unlikely that more RCTs will show any significant impact of vitamin D as a cardioprotective intervention.

Our meta-analysis has many strengths. It has very low heterogeneity in all of the outcomes, increased sample size by almost 30%, and increased power of the statistical analysis compared to the previous meta-analyses. Moreover, we registered our meta-analysis on the PROSPERO database and followed a rigorous methodology.

Vitamin D supplementation can cause different adverse effects when taken in doses greater than 4000 IU daily. These include hypercalcemia, hypercalcuria, constipation, confusion, disorientation, and arrhythmias.

Our research also has some limitations. As it is a meta-analysis, we are considering study-level data, not patient-level data. Four RCTs were judged to be at a medium risk of bias. The follow-up periods of the individual RCTs were not very long, which could deter us from detecting some potential beneficial cardiovascular effects. Most of the trials did not have cardiovascular outcomes as a primary outcome. Also, very few trials reported data on heart failure and coronary revascularization, causing their results to be underpowered. The definition of MACE also varied a lot between different trials. The limited follow-up periods of individual studies may have prevented the detection of potential cardiovascular effects of vitamin D supplementation. The medium risk of bias in some included studies may also impart some bias in our findings.

There are many implications of our meta-analysis. Regarding clinical implications, our meta-analysis supports the existing literature that vitamin D does not confer a benefit to people regarding their cardiovascular health and, thus, should not be used for this purpose. Moreover, our meta-analysis suggests that more RCTs with longer follow-ups are needed to better detect any cardiovascular effects. The RCTs should also be designed through risk factor optimization to include patients with an increased risk of cardiovascular disease.

## Conclusion

According to our meta-analysis, vitamin D supplementation did not reduce major adverse cardiovascular events, other cardiovascular parameters, and all-cause mortality. Therefore, vitamin D should not be recommended for the prevention of cardiovascular disease.

## Ethical approval

No ethical approval was required for this study.

## Consent

No consent was required for this study.

## Source of funding

No financial support was received for this study.

## Author contribution

M.A., A.M.W.M., N.E.A., and M.F.M.: study conception and design; A.M.W.M., N.E.A., M.F.M., and T.B.: study conduct and acquisition of data; A.M.W.M., N.E.A., M.F.M., S.V.U., and A.A.: data analysis; M.A., N.E.A., M.F.M., T.B., S.L., and I.W.F.: data interpretation; A.M.W.M., N.E.A., M.A., M.F.M., T.B., and S.V.U.: drafting of the manuscript; M.A., I.W.F., S.L., H.F., A.A., R.M., and M.K.: critical revision of the manuscript. Final approval of the version was approved by all the authors to be published. All authors agree to be accountable for all aspects of the work.

## Conflicts of interest disclosure

The authors declare no conflicts of interest.

## Research registration unique identifying number (UIN)

PROSPERO registration number: CRD42023458654.

## Guarantor

Kamal Kandel.

## Data availability statement

The data that support the findings of this study are available from the corresponding author, K.K., upon reasonable request.

## Provenance and peer review

Not applicable.

## Supplementary Material
