# Supplementary material for: Effect of vitamin D supplementation on cardiovascular outcomes: an updated meta-analysis of RCTs
Source: Ann Med Surg (Lond). 2024 Aug 14;86(11):6665–72. doi: 10.1097/MS9.0000000000002458 (PMC11543226; doi:10.1097/MS9.0000000000002458)

**Supplementary Material**

**Effect of Vitamin D supplementation on cardiovascular outcomes: an updated meta-analysis of RCTs**

**Authors**

Agha Muhammad Wali Mirza^1^, Naiela Ennaji Almansouri^2^, Muhammad Fareed Muslim^3^, Thahaseen Basheer^4^, Sree Varuntej Uppalapati^5^, Swati Lakra^6^, Hareem Fatima^7^, Arshiya Adhnon^8^, Ivo Wandark Filho^9^, Ruqeyya Mahmood^10^, Mahendra Kumar^11^, Muhammad Ayyan^12*^

**Authors’ affiliations**

^1^ Department of Internal Medicine, Jinnah Medical & Dental College, Karachi, Pakistan

^2^ Department of Internal Medicine, University of Tripoli Faculty of Medicine, Tripoli, Libya

^3^ Department of Internal Medicine, Allama Iqbal Medical College, Lahore, Pakistan

^4^ Department of Internal Medicine, Karuna Medical College, Kerala, India

^5^ Department of Internal Medicine, All American Institute of Medical Sciences, Jamaica

^6^ Department of Internal Medicine, Holy Heart Hospital, Rohtak, India

^7^ Department of Internal Medicine, Federal Medical College, Islamabad, Pakistan

^8^ Department of Internal Medicine, Dubai Medical College for Girls, Dubai, United Arab Emirates

^9^ Department of Internal Medicine, Faculdade Pernambucana de Saúde (FPS), Recife, Brazil

^10^ Department of Internal Medicine, Deccan College of Medical Sciences, Hyderabad, India

^11^ Department of Internal Medicine, Sardar Patel Medical College, Bikaner, India

^12^ Department of Internal Medicine, Mayo Hospital, Lahore, Pakistan

***Corresponding Author:**

Muhammad Ayyan

**Email:** [iamayyan77@gmail.com](mailto:iamayyan77@gmail.com)

**Address:** Department of Medicine, Mayo Hospital, Nila Gumbad Chowk, Neela Gumbad Lahore, Punjab, Pakistan 54000

**Telephone:** +92 3238478847

**Twitter handle:** @ayyan_77

**Keywords:** meta-analysis; Vitamin D; cardiovascular; cardioprotective; cardiology

**Acknowledgments:** N/A

**Declarations of interest**

The authors declare that they have no conflicts of interest and no financial interests related to the material of this manuscript.

**Supplementary Figure S1.1.** Comparison of incidence of Myocardial Infarction between patients receiving Vitamin D or control. IV, inverse variance. MACE, Major Adverse Cardiovascular Events.

**Supplementary Figure S1.2.** Comparison of incidence of Cerebrovascular accident between patients receiving Vitamin D or control. IV, inverse variance. MACE, Major Adverse Cardiovascular Events.

**Supplementary Figure S1.3.** Comparison of incidence of Heart failure between patients receiving Vitamin D or control. IV, inverse variance. MACE, Major Adverse Cardiovascular Events.

**Supplementary Figure S1.4.** Comparison of incidence of Coronary revascularization between patients receiving Vitamin D or control. IV, inverse variance. MACE, Major Adverse Cardiovascular Events.

**Supplementary Figure S1.5.** Risk of bias assessment of RCTs using RoB 2.0 scale.

**Supplementary Figure S1.1.** Comparison of incidence of Myocardial Infarction between patients receiving Vitamin D or control. IV, inverse variance. MACE, Major Adverse Cardiovascular Events.


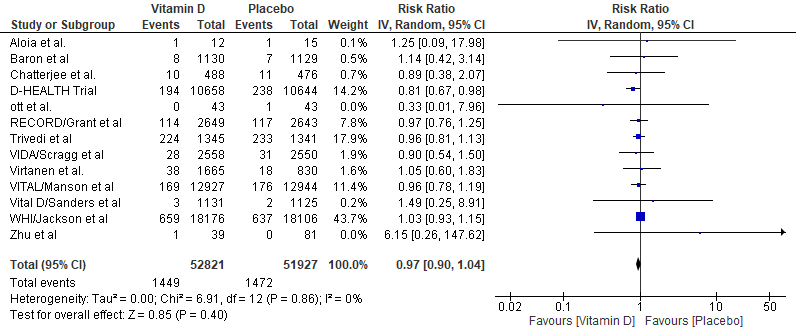


**Supplementary Figure S1.2.** Comparison of incidence of Cerebrovascular accident between patients receiving Vitamin D or control. IV, inverse variance. MACE, Major Adverse Cardiovascular Events.


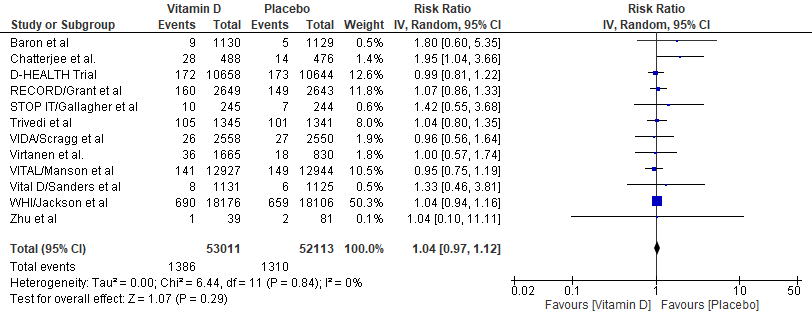


**Supplementary Figure S1.3.** Comparison of incidence of Heart failure between patients receiving Vitamin D or control. IV, inverse variance. MACE, Major Adverse Cardiovascular Events.


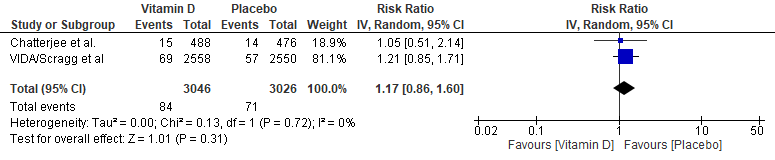


**Supplementary Figure S1.4.** Comparison of incidence of Coronary revascularization between patients receiving Vitamin D or control. IV, inverse variance. MACE, Major Adverse Cardiovascular Events.


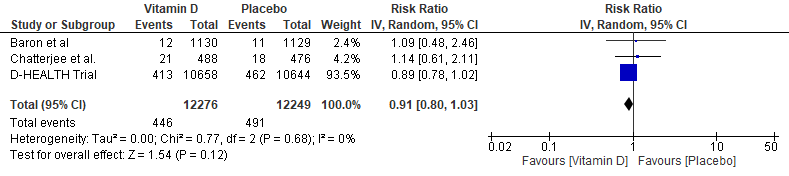


**Supplementary Figure S1.5.** Risk of bias assessment of RCTs using RoB 2.0 scale.


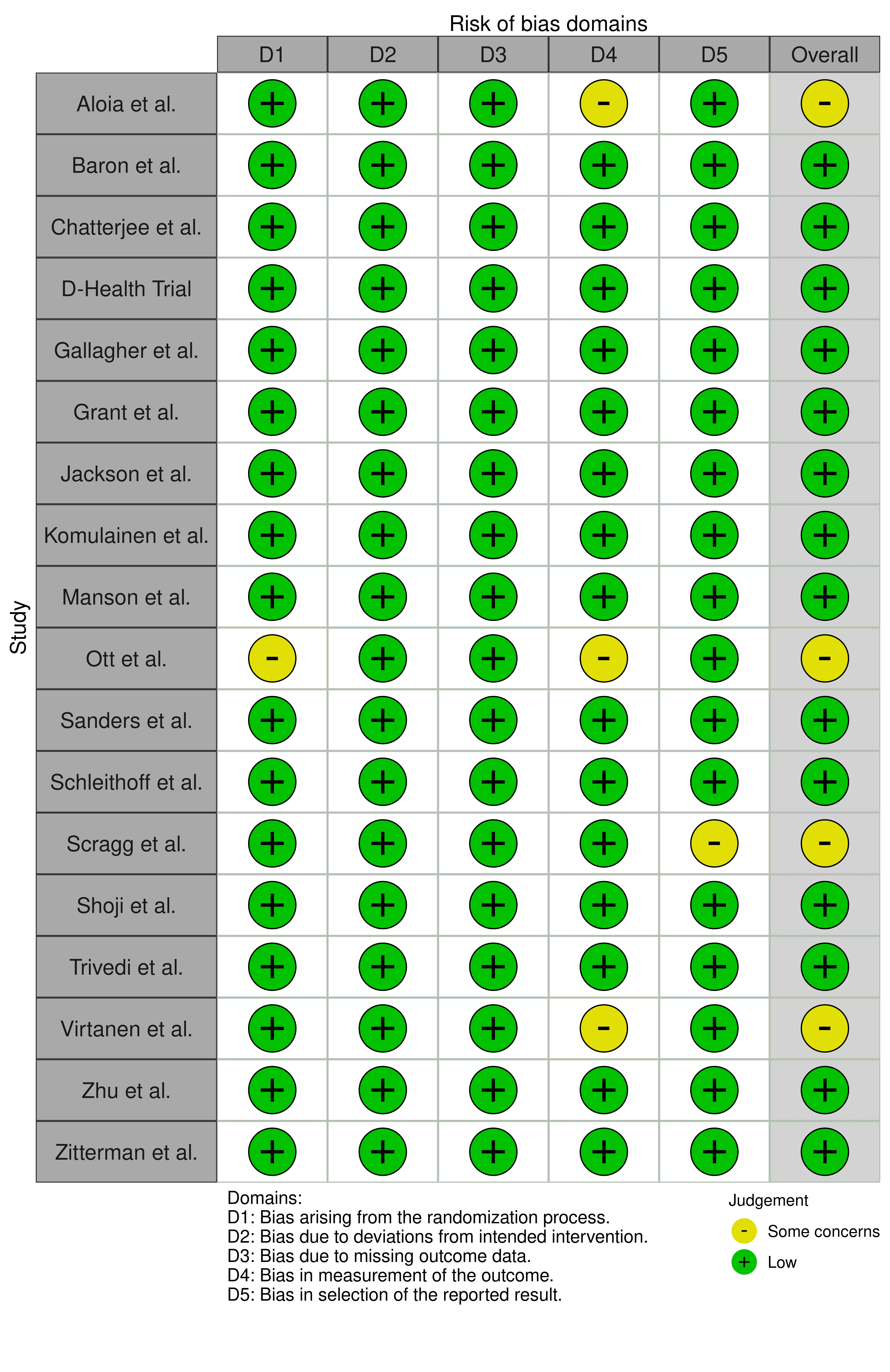

Supplement: Supplementary file 3 [file ms9-86-6665-s003.docx]
